# Posttraumatic aortic regurgitation and root pseudoaneurysm following blunt chest trauma: a case report

**DOI:** 10.1186/s40792-024-01963-1

**Published:** 2024-07-31

**Authors:** Hiroaki Aizawa, Haruo Yamauchi, Masahiko Ando, Minoru Ono

**Affiliations:** Department of Cardiovascular Surgery, The University of Tokyo Hospital, 7-3-1 Hongo, Bunkyo, Tokyo 113-8655 Japan

**Keywords:** Posttraumatic aortic regurgitation, Root pseudoaneurysm, Marfan syndrome

## Abstract

**Background:**

The simultaneous diagnosis of severe aortic regurgitation and aortic root pseudoaneurysm resulting from traffic injury is extremely rare. This report presents the case of a patient with Marfan syndrome who experienced aortic root pseudoaneurysm and subacute severe aortic regurgitation following a traffic accident.

**Case presentation:**

A 64-year-old woman was diagnosed with Marfan syndrome 16 years ago and is undergoing ongoing follow-up at an outpatient clinic. Eight years previously, the patient underwent total arch replacement combined with J-graft open stent graft^®^ (JGOS; Japan Lifeline Co., Tokyo, Japan) deployment for acute type A dissection. Five months before presentation, the patient suffered a left rib fracture in a traffic accident and received conservative management at a local hospital. The patient presented to the emergency room with worsening shortness of breath and orthopnea. Echocardiography revealed severe aortic regurgitation and moderate tricuspid regurgitation. Computed tomography revealed new-onset pseudoaneurysm at the aortic root. Surgical repair was successfully performed using a modified Bentall procedure with a bioprosthetic valve and tricuspid annuloplasty. Intraoperative findings revealed pseudoaneurysm with perforation of the right sinus of Valsalva. Although the left and right aortic valve leaflets were normal, the noncoronary leaflet exhibited a ruptured fibrous strand of a cusp fenestration, resulting in acute aortic regurgitation.

**Conclusions:**

This case report highlights the rare occurrence of aortic root pseudoaneurysm and subacute aortic regurgitation following a traffic accident. In cases of blunt chest trauma, particularly in patients with Marfan syndrome, frequent examination is crucial to assess the possibility of posttraumatic aortic regurgitation and aortic injury.

## Background

Blunt aortic root injury resulting from a traffic accident is a rare disorder. Nonpenetrating traumatic injury of the heart primarily affects the right heart system, with the aortic valve being the most commonly affected valve leaflet and the aortic isthmus being the most frequently injured part of the aorta [1, 2]. However, the simultaneous occurrence of severe aortic regurgitation (AR) and aortic root pseudoaneurysm following blunt trauma due to a traffic accident is extremely rare. This report presents a successful case of surgical intervention for subacute severe AR and root pseudoaneurysm occurring 5 months after blunt trauma in a patient with Marfan syndrome.

## Case presentation

A 64-year-old woman diagnosed with Marfan syndrome 16 years previously experienced acute type A aortic dissection with an intimal tear located under the origins of the brachiocephalic and left subclavian arteries 8 years previously. The false lumen at the aortic root was filled with thrombi. The patient underwent emergent surgery of total arch replacement using a J-graft open stent graft^®^ (JGOS) (Japan Lifeline Co., Tokyo, Japan). Intraoperative findings revealed no abnormalities of the aortic valve cusps. We removed the thrombus from the false lumen of the aortic root and sealed it using a surgical adhesive (BioGlue^®^; CryoLife Inc., Kennesaw, GA, USA). The patient was discharged without any major complications. Postoperative genetic testing confirmed *FBN1* gene abnormality. One year previously, periodic echocardiography revealed unchanged mild AR and an enlarged Valsalva sinus of 41 mm. No other abnormal findings of the aortic root were observed on computed tomography (CT) (Fig. 2a). Five months previously, the patient had a collision with a bicycle while walking, resulting in a left rib fracture, and received conservative management at a local hospital. One month previously, the patient complained of occasional shortness of breath; however, chest X-ray showed a cardiothoracic ratio of 51.5% without a sign of pulmonary congestion, pleural effusion, or pneumothorax. One week previously, the patient exhibited worsening shortness of breath upon exertion and orthopnea and was admitted to our hospital.

**Fig. 1:**
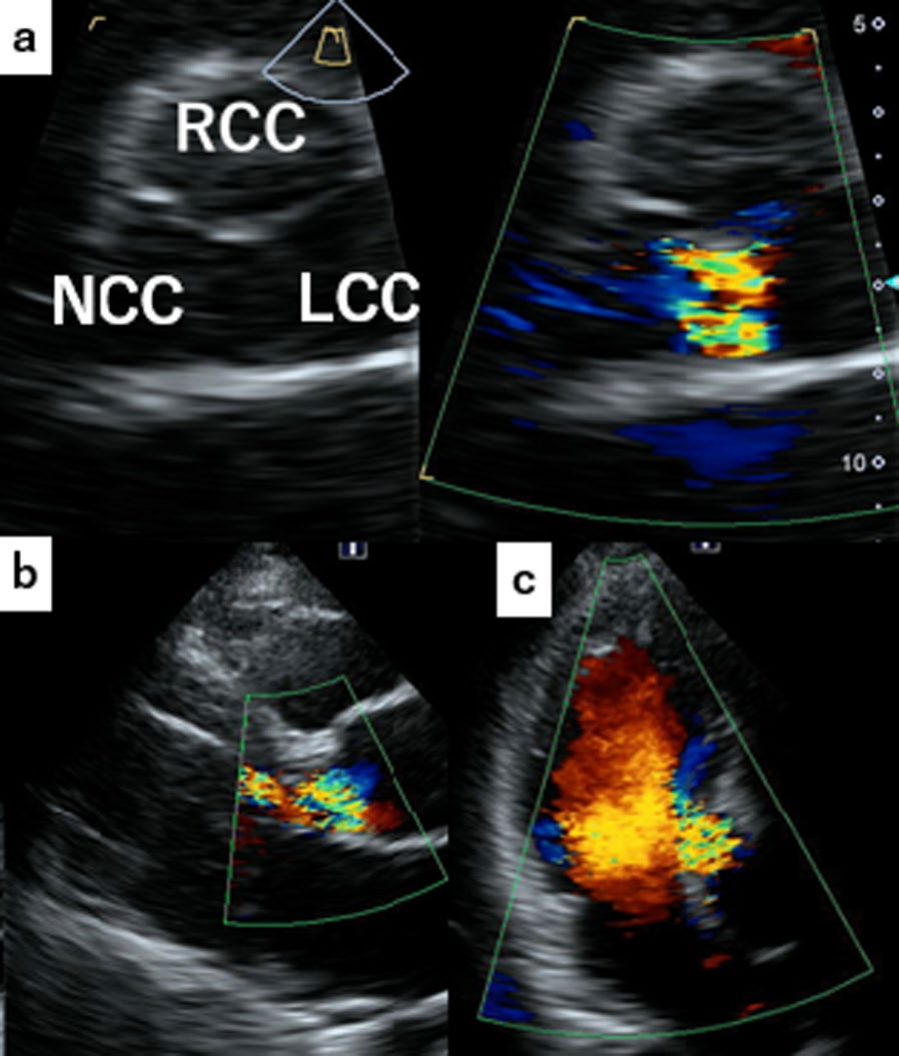
Echocardiographic findings of aortic valve and regurgitation. Transthoracic echocardiography in parasternal short-axis (**a**), long-axis (**b**), and three-chamber (**c**) views. The jet of severe aortic regurgitation is directed toward the left ventricle between the noncoronary cusp (NCC) and the left coronary cusp (LCC). The right coronary cusp (RCC) is also indicated

**Fig. 2:**
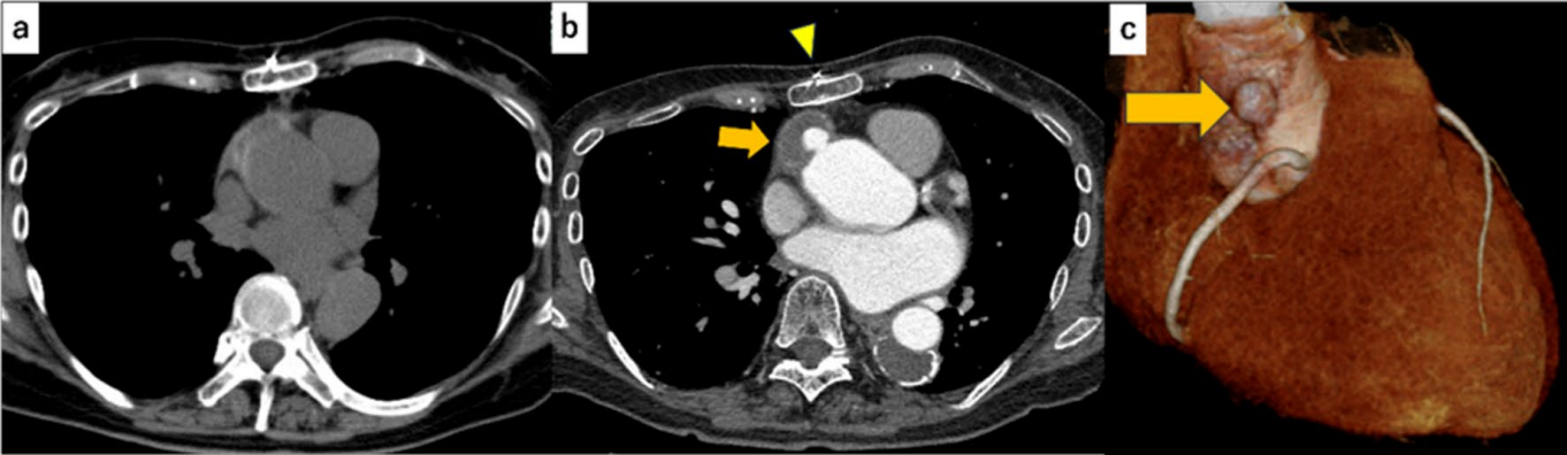
Computed tomographic images of aortic root. Pre-trauma (**a**) and post-trauma (**b**) axial computed tomographic images, and a lateral view of the Valsalva sinus in three-dimensional volume-rendered cardiac computed tomography (**c**). Arrows indicate pseudoaneurysm of the aortic root. An arrowhead indicates a sternal wire

On physical examination, blood pressure was 142/66 mmHg, pulse rate was 85 beats/min, and peripheral oxygen saturation (SpO_2_) was 98% (on nasal 3 L/min of oxygen). Auscultation revealed a Levine Grade III diastolic regurgitation murmur at the left sternal border. Electrocardiogram revealed no abnormal findings, and chest X-ray indicated a cardiothoracic ratio of 59.9% with severe pulmonary congestion and bilateral pleural effusion. The brain natriuretic peptide level was 599.6 pg/mL. Echocardiography revealed a left ventricular ejection fraction of 62%, left ventricular end-diastolic/end-systolic dimensions of 56/37 mm, severe AR (Fig. 1), and moderate tricuspid regurgitation. The aortic valve had tricuspid leaflets with an aortic annulus of 24 mm; the diameters of the Valsalva sinus and sinotubular junction were 41 and 37 mm, respectively. Because left ventricular enlargement was not prominent, rapid progression of AR was suspected. CT performed after the trauma revealed an unchanged Valsalva sinus size, although a 17-mm pseudoaneurysm was newly observed on the anterior side of the aortic root adjacent to the sternal wire (Fig. 2b and c).

**Fig. 3:**
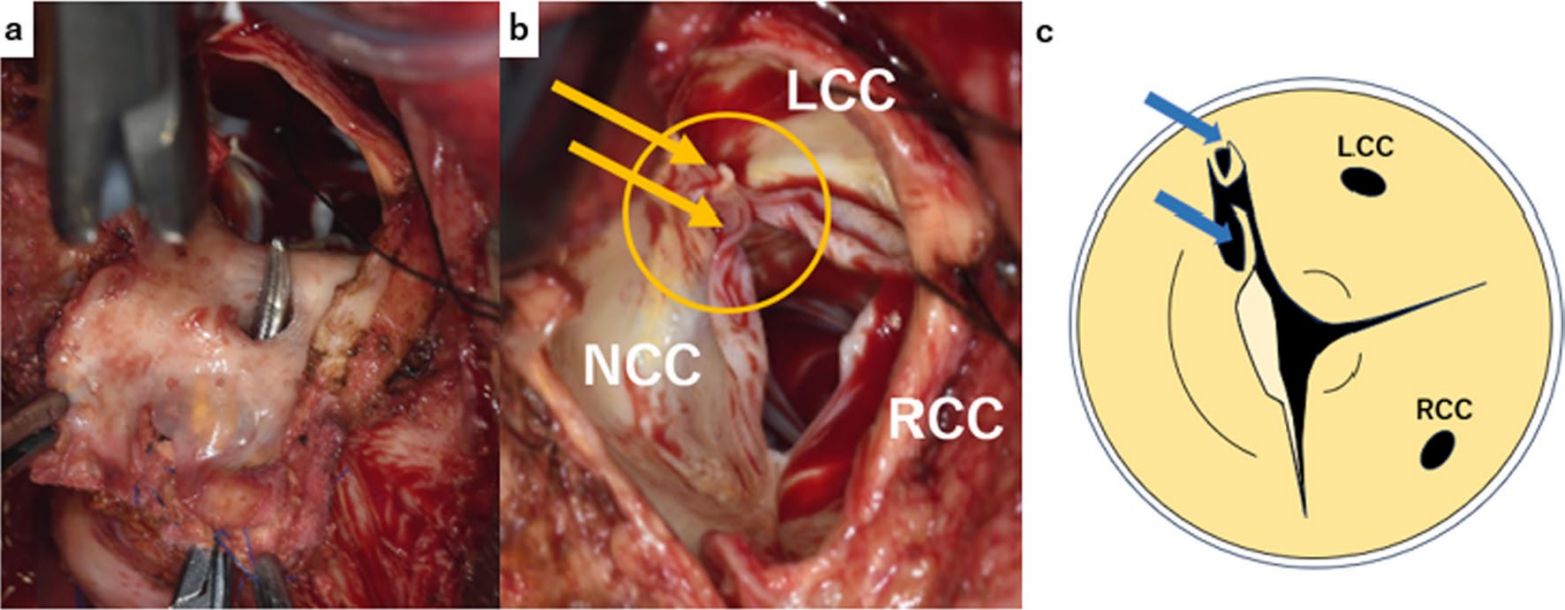
Intraoperative findings of aortic root and valve. A 7-mm pseudoaneurysmal entry was observed in the right sinus of Valsalva (**a**). The noncoronary cusp (NCC) was fenestrated (circle) and exhibited a ruptured fibrous strand (arrow) (**b**). The left coronary cusp (LCC) and right coronary cusp (RCC) are also indicated. Schema of the ruptured fibrous strand (arrow) (**c**)

Heart failure management was provided for 2 weeks. After redo-median sternotomy, aortic cross-clamping, and cardiac arrest, the ascending aorta was opened under total cardiopulmonary bypass. A 7-mm perforation was observed on the anterior side of the Valsalva sinus (Fig. 3a). Although the left and right aortic valve leaflets had no abnormalities, the noncoronary leaflet exhibited fenestration with a ruptured fibrous strand originating from the left-noncoronary commissure (Fig. 3b and c), which was identified as the cause of noncoronary cusp prolapse. We performed a modified Bentall procedure using Gelweave Valsalva Graft^®^ 28 mm (Terumo Corporation, Tokyo, Japan) and a 25-mm INSPIRIS RESILIA^®^ valve (Edwards Lifesciences LLC, Irvine, CA, USA). Both coronary artery buttons were anastomosed onto the Valsalva Graft^®^ using the Carrel patch technique. The distal portion of the Valsalva Graft^®^ was properly trimmed and anastomosed to the previous total arch graft. Furthermore, no obvious abnormalities of the tricuspid valve leaflets, such as leaflet prolapse due to injured chordae tendineae or leaflet rupture, were observed. Tricuspid regurgitation was caused by dilation of the tricuspid valve annulus, suggesting that the etiology of tricuspid regurgitation was not primary but secondary to AR. Therefore, a 28-mm Carpentier–Edwards Physio-Tricuspid Annuloplasty Ring^®^ (Edwards Lifesciences LLC, Irvine, CA, USA) was applied. Weaning from cardiopulmonary bypass was uneventful. The cardiopulmonary bypass time was 239 min, and the aortic cross-clamp time was 149 min. Pathological examination revealed mucoid degeneration of the aortic valve cusps. The media’s elastic fibers were degenerated and replaced with fibrous tissues in the pseudoaneurysm region of the aorta. The patient’s postoperative course was uneventful, and the patient was discharged 3 weeks after surgery. Eight months after the operation, the patient was in good health.

## Discussion

Blunt cardiac traumas account for < 10% of all trauma admissions [3]. Such trauma is commonly caused by motor vehicle accidents, pedestrian incidents, and falls from heights [3]. Among blunt cardiac trauma cases, aortic valve injuries are the most common, followed by mitral, tricuspid, and pulmonary valve injuries [3]. Posttraumatic AR following blunt chest trauma is thought to result from a sudden pressure increase during the early diastolic phase, when the transaortic pressure gradient peaks [4]. Among the three aortic valve cusps, the noncoronary cusp is the most vulnerable because it lacks a coronary artery opening and acts as a buffer for increased intra-aortic pressure [4]. Posttraumatic AR can occur not only because of direct valve injury but also through the progression of ascending aortic wall dissection, extending to the commissural and valve attachment areas [5].

In this case, acute AR was caused by the rupture of fenestrated fibrous strands, suggesting direct aortic valve cusp injury as the primary cause. Aortic valve fenestrations are defined as the loss of tissue in aortic valve leaflets, which occurs in all age groups, and their size increases with aging [6]. They are mainly located at the free edge of the leaflet at the commissural area, which is embryologically the weakest point of leaflet tissue [6]. Lifelong hemodynamical forces, mechanical stress caused by the dilation of the aortic root, sudden trauma, and iatrogenic injury causes fenestration formation [6]. Enlarged fenestration and fibrous strand rupture can lead to massive regurgitation and result in acute left heart failure [7].

Aortic valve cusp commissural fenestrations are well-described features of Marfan syndrome because of tissue fragility. Although large fenestrations as a cause of significant AR are rarely observed because patients with Marfan syndrome tend to receive early surgery nowadays [8], AR due to spontaneous rupture of a fenestrated fibrous strand in Marfan syndrome has been reported [9]. Pathological examination in this case revealed myxomatous degeneration of the valve leaflets, which has been reported as an important pathogenetic characteristic of fenestration-related massive AR [10].

A previous study analyzing 95 cases of posttraumatic AR reported that 69% of the cases were diagnosed within 3 months of onset, and approximately 5% were diagnosed > 5 years after trauma; thus, the time span from trauma to the onset of symptoms and diagnosis varies widely [11]. Although a fibrous strand at the commissural area might have been ruptured at the time of trauma in this case, the involved aortic cusp with a ruptured site retained some compensatory coaptation zone in the diastole phase [7], and AR was not severe at the time of trauma. The hemodynamic stress increased over the involved aortic cusp with worsening AR. Cusp remodeling progressed and finally led to a remarkable cusp prolapse [12]. Generally, posttraumatic AR is primarily treated with aortic valve replacement, expecting quick and definitive treatment effects, considering the urgency of surgery based on deteriorated cardiac function [4]. Valve repair may be an option if degeneration is limited to a single cusp and the overall health condition is accepted, although caution is warranted because microscopic trauma-induced degeneration in an apparently normal valve leaflet can lead to the postoperative recurrence of AR [11].

Blunt aortic injuries tend to be a fatal condition, with 80% of patients dying before presentation and diagnosis, and they most commonly occur in the aortic isthmus and ascending aorta [2, 13]. These injuries result from mechanical factors, such as shear, torsion, pinch, stretch, and hydrostatic forces, acting on the aortic wall [2, 14]. Blunt aortic injuries can cause pseudoaneurysms depending on the injured aortic tissue conditions and past aortic surgeries [2, 14]. Pseudoaneurysms are anatomically classified into four grades based on the aortic wall layers: intimal tear (Grade I), intramural hematoma (Grade II), pseudoaneurysm (Grade III), and rupture (Grade IV) [15]. Grade I and II cases may be managed nonoperatively through blood pressure control and imaging surveillance [16]. In Grade III cases, urgent or emergency surgeries may be required, depending on the patient’s condition and associated injuries. Grade IV cases require emergency surgeries, considering both surgical and endovascular interventions [2, 16]. The pseudoaneurysm of the aortic root in this case corresponded to Grade III. Pathologically, the pseudoaneurysm site exhibited evidence of the loss of tunica media elastic fibers in the aortic wall, which is consistent with the pathological features of Marfan syndrome. Aortic root replacement or repair is highly recommended for the emergent surgery for type A dissection in patients with Marfan syndrome because supracoronary ascending replacement is associated with a high need for root reintervention because of aortic insufficiency and root aneurysm [17]. However, in our case, we did not select aortic root replacement during the initial surgery because Marfan syndrome was recognized after the first surgery by genetic testing. Moreover, the false lumen of the aortic root was sealed with BioGlue^®^ during the first surgery. Because BioGlue^®^-induced tissue toxicity has been reported [18], it could also have contributed to the formation of pseudoaneurysms in the aortic root in addition to the traumatic impact. Previously, pseudoaneurysms of the ascending aorta caused by fractured sternal wires have been reported [19]. In our case, although no evidence of sternal wire fracture was observed, the wire was positioned adjacent to the pseudoaneurysm orifice. Thus, the sternal wires may have contributed to adventitial injuries and intimal tears of the aortic root during trauma.

The simultaneous occurrence of severe AR and aortic root pseudoaneurysm following blunt trauma is extremely rare [20]. To the best of our knowledge, a case of the simultaneous occurrence of severe AR and aortic root pseudoaneurysm in a patient with Marfan syndrome has never been reported.

## Conclusions

We presented a case involving simultaneous aortic root pseudoaneurysm and subacute AR following blunt chest trauma. When a patient with Marfan syndrome presents with blunt chest traumas, frequent examination is essential even without a symptom, considering the possible blunt aortic and/or aortic valve injury.

## Data Availability

Not applicable.

## Abbreviations

CT: Computed tomography
AR: Aortic regurgitation

## References

[1] Nair L, Winkle B, Senanayake E. Managing blunt cardiac injury. J Cardiothorac Surg. 2023;18:71. 36765392 10.1186/s13019-023-02146-z PMC9912488

[2] Akhmerov A, DuBose J, Azizzadeh A. Blunt thoracic aortic injury: current therapies, outcomes, and challenges. Ann Vasc Dis. 2019;12:1–5. 30931049 10.3400/avd.ra.18-00139 PMC6434345

[3] El-Andari R, O’Brien D, Bozso SJ, Nagendran J. Blunt cardiac trauma: a narrative review. Mediastinum. 2021;5:28. 35118333 10.21037/med-21-19 PMC8799926

[4] Li W, Ni Y, Chen X, Ma L. Aortic valve tear with severe aortic regurgitation following blunt chest trauma. J Cardiothorac Surg. 2011;6:84. 21682925 10.1186/1749-8090-6-84 PMC3133542

[5] Matteucci ML, Rescigno G, Altamura G, Manfrin M, D’Alfonso A, Piccoli G, Iacobone G. Delayed traumatic aortic cusp detachment mimicking aortic dissection. Ann Thorac Surg. 2006;82:1093–5. 16928547 10.1016/j.athoracsur.2006.01.030

[6] Dudkiewicz D, Zhingre Sanchez JD, Hołda J, Bolechała F, Strona M, Kopacz P, et al. Aortic valve fenestrations: macroscopic assessment and functional anatomy study. Clin Anat. 2023;36:612–7. 36597994 10.1002/ca.24002

[7] Ishige A, Uejima T, Kanmatsuse K, Endo M. Giant fenestration and fibrous strand rupture of aortic valve without massive regurgitation. J Cardiol Cases. 2012;5:e163–5. 30532930 10.1016/j.jccase.2012.02.008 PMC6265424

[8] Mastrobuoni S, Tamer S, Navarra E, de Kerchove L, El Khoury G. Aortic valve repair in patients with Marfan syndrome-the “Brussels approach.” Ann Cardiothorac Surg. 2017;6:704–8. 29270384 10.21037/acs.2017.10.01 PMC5721118

[9] Akiyama K, Ohsawa S, Hirota J, Takiguchi M. Massive aortic regurgitation by spontaneous rupture of a fibrous strand in a fenestrated aortic valve. J Heart Valve Dis. 1998;7:521–3. 9793850

[10] Akiyama K, Hirota J, Taniyasu N, Maisawa K, Kobayashi Y, Tsuda M. Pathogenetic significance of myxomatous degeneration in fenestration-related massive aortic regurgitation. Circ J. 2004;68:439–43. 15118285 10.1253/circj.68.439

[11] Tsugu T, Murata M, Mahara K, Iwanaga S, Fukuda K. Long-term survival on medical therapy alone after blunt-trauma aortic regurgitation: report of a new case with summary of 95 others. Tex Heart Inst J. 2016;43:446–52. 27777534 10.14503/THIJ-15-5151 PMC5067044

[12] Noda K, Takahashi Y, Morisaki A, Sakon Y, Nishiya K, Inno G, et al. Delayed traumatic aortic valve perforation after blunt chest trauma. Surg Case Rep. 2024;10:39. 38353758 10.1186/s40792-024-01837-6 PMC10866806

[13] Neschis DG, Scalea TM, Flinn WR, Griffith BP. Blunt aortic injury. N Engl J Med. 2008;359:1708–16. 18923173 10.1056/NEJMra0706159

[14] Richens D, Field M, Neale M, Oakley C. The mechanism of injury in blunt traumatic rupture of the aorta. Eur J Cardiothorac Surg. 2002;21:288–93. 11825737 10.1016/S1010-7940(01)01095-8

[15] Azizzadeh A, Keyhani K, Miller CC 3rd, Coogan SM, Safi HJ, Estrera AL. Blunt traumatic aortic injury: initial experience with endovascular repair. J Vasc Surg. 2009;49:1403–8. 19497498 10.1016/j.jvs.2009.02.234

[16] De Freitas S, Joyce D, Yang Y, Dunphy K, Walsh S, Fatima J. Systematic review and meta-analysis of nonoperative management for SVS grade II blunt traumatic aortic injury. Ann Vasc Surg. 2024;98:220–7. 37806657 10.1016/j.avsg.2023.07.106

[17] Rylski B, Bavaria JE, Beyersdorf F, Branchetti E, Desai ND, Milewski RK, Szeto WY, Vallabhajosyula P, Siepe M, Kari FA. Type A aortic dissection in Marfan syndrome: extent of initial surgery determines long-term outcome. Circulation. 2014;129:1381–6. 24594630 10.1161/CIRCULATIONAHA.113.005865

[18] Luk A, David TE, Butany J. Complications of Bioglue postsurgery for aortic dissections and aortic valve replacement. J Clin Pathol. 2012;65:1008–12. 22872707 10.1136/jclinpath-2012-200809

[19] Amirghofran AA, Nirooei E, Ostovan MA. Ascending aorta graft pseudoaneurysm and aortobronchial fistula caused by a fractured sternal wire: a case report. J Cardiothorac Surg. 2021;16(1):348. 34876204 10.1186/s13019-021-01737-y PMC8649677

[20] de Castro D, Rasines-Rodríguez A, Usano A, Mingo S. Acute post-traumatic aortic regurgitation. JACC Case Rep. 2022;4:1432–4. 36388713 10.1016/j.jaccas.2022.07.018 PMC9663896

